# Rethinking children’s physical activity interventions at school: A new context-specific approach

**DOI:** 10.3389/fpubh.2023.1149883

**Published:** 2023-04-13

**Authors:** Russell Jago, Ruth Salway, Danielle House, Michael Beets, David Revalds Lubans, Catherine Woods, Frank de Vocht

**Affiliations:** ^1^Centre for Exercise, Nutrition and Health Sciences, School for Policy Studies, University of Bristol, Bristol, United Kingdom; ^2^Population Health Sciences, Bristol Medical School, University of Bristol, Bristol, United Kingdom; ^3^The National Institute for Health Research, Applied Research Collaboration West (NIHR ARC West), University Hospitals Bristol and Weston NHS Foundation Trust, Bristol, United Kingdom; ^4^NIHR Bristol Biomedical Research Centre, University Hospitals Bristol and Weston NHS Foundation Trust and University of Bristol, Bristol, United Kingdom; ^5^Arnold School of Public Health, University of South Carolina, Columbia, SC, United States; ^6^Centre for Active Living and Learning, College of Human and Social Futures, University of Newcastle, Callaghan, NSW, Australia; ^7^Hunter Medical Research Institute, Newcastle, NSW, Australia; ^8^Faculty of Sport and Health Sciences, University of Jyväskylä, Jyväskylä, Finland; ^9^Physical Activity for Health Research Centre, Health Research Institute, University of Limerick, Limerick, Ireland

**Keywords:** physical activity, children, trial, context, intervention—behavioral

## Abstract

Physical activity is important for children’s health. However, evidence suggests that many children and adults do not meet international physical activity recommendations. Current school-based interventions have had limited effect on physical activity and alternative approaches are needed. Context, which includes school setting, ethos, staff, and sociodemographic factors, is a key and largely ignored contributing factor to school-based physical activity intervention effectiveness, impacting in several interacting ways.

**Conceptualization:**

Current programs focus on tightly-constructed content that ignores the context in which the program will be delivered, thereby limiting effectiveness. We propose a move away from uniform interventions that maximize internal validity toward a flexible approach that enables schools to tailor content to their specific context.

**Evaluation designs:**

Evaluation of context-specific interventions should explicitly consider context. This is challenging in cluster randomized controlled trial designs. Thus, alternative designs such as natural experiment and stepped-wedge designs warrant further consideration.

**Primary outcome:**

A collective focus on average minutes of moderate-to-vigorous intensity physical activity may not always be the most appropriate choice. A wider range of outcomes may improve children’s physical activity and health in the long-term. In this paper, we argue that greater consideration of school context is key in the design and analysis of school-based physical activity interventions and may help overcome existing limitations in the design of effective interventions and thus progress the field. While this focus on context-specific interventions and evaluation is untested, we hope to stimulate debate of the key issues to improve future physical activity intervention development and implementation.

## 1. Introduction

Despite their considerable potential, current school-based interventions for children and young people have had limited effect on device-measured physical activity, and have not met their primary aim of increasing physical activity at a population level (1–3). A key challenge for the field is therefore to identify why these interventions have not yielded the hypothesized impacts and how they could be improved. In this paper we argue that context, which includes a combination of school setting, ethos, staff, and sociodemographic factors, is a key and largely ignored contributing factor to physical activity intervention effectiveness (4). Context impacts on the effectiveness of interventions in several interacting ways. The first is the way in which interventions are conceptualized. Most school-based physical activity interventions are tightly-constructed programs that fail to take account of the context in which the program will be delivered. This failure to take account of the context negatively affects generalizability and scalability. Second is ignoring context in the evaluation. We need to find a better trade-off between optimizing internal validity and understanding which interventions are effective and in which contexts. The third, often integrated, element is the focus on average minutes of moderate-to-vigorous intensity physical activity (MVPA) as the primary outcome, which may not always be the most appropriate choice or indicator of success. To address these issues, this paper first defines what we mean by context and outlines why failure to address context has hindered our ability to increase children’s physical activity. We then propose an alternative **context-specific approach** to intervention design, in which the context informs the intervention content, choice of outcomes, evaluation design, and analysis. Finally, we provide an example of a forthcoming project that uses a context-specific design, the challenges that the study poses and how we intend to address them.

## 2. What is context?

Context has been conceptualized as a “a set of characteristics and circumstances that consist of active and unique factors that surround the implementation” of an intervention and its evaluation (4). This includes the cultural, social, economic, political, and/or organizational setting as well as the demographic, epidemiological, and socioeconomic characteristics of those delivering and receiving the physical activity intervention (5, 6). For school-based physical activity interventions, this includes the setting of the school, the demographic profile of the pupils, the facilities available, the attitudes, training and skill of school staff as well as school priorities and the interests of the children. It is important to note that some aspects, such as priorities, attitudes and training, are amenable to change, while others, such as school size, location, and pupil demographics, are fixed constraints.

## 3. Why is physical activity important for children and young people?

Physical activity is associated with improved physical and mental health across the life course (7, 8). Among children, physical activity is associated with lower levels of risk factors such as cholesterol and blood lipids, favorable blood pressure, and lower adiposity (9). These risk factors are more prevalent in children with a lower socioeconomic position (10). Physical activity is also associated with improved well-being, self-esteem, and academic performance in young people (11). Physical activity tracks from childhood into adulthood, with more active children likely to engage in both a higher amount and wider range of physical activities in adulthood (12, 13).

The World Health Organization recommends that children and young people engage in an average of 60 min of MVPA per day that can be accumulated across the day (7, 14). International collaborations and national surveys indicate, however, that many children and young people do not meet the current physical activity guidelines (15–17). Physical activity levels decline during childhood and adolescence with a steeper decline for girls than boys (16, 17). For example, data from around 2,000 children from 57 schools in the Bristol B-Proact1v cohort begun in 2012/13 showed that mean minutes of MVPA per day on weekdays declined by 2.2 min per year (95% confidence interval 1.9 to 2.5), between 6 and 11 years of age. National lockdowns during the COVID-19 pandemic resulted in acute changes to physical activity opportunity and provision, and emerging data show that physical activity and mental well-being declined during this time (18–22). The impacts of COVID-19 were more marked for children from lower socio-economic backgrounds and those without access to outdoor space (20, 23). Collectively, these data highlight a need to increase physical activity and prevent the age-related decline among children and young people.

## 4. How effective are current school-based approaches to increase children’s physical activity?

Most attempts to increase physical activity among children and young people have been delivered at the school site, as schools provide opportunities to implement universal public health interventions to large numbers of children (24). Although there has been recognition that whole-school physical activity interventions, in which more than one element of the school physical activity provision is changed, hold considerable promise (1), most of the intervention literature describes single-component interventions (e.g., changing aspects of physical education) (2, 3). Meta-analyses have shown that, with a small number of exceptions, these programs have not yielded increases in MVPA (2, 3, 25–27). Often the “failure” of these interventions can be attributed to implementation issues such as the failure to deliver the program as intended, poor attendance, or lack of access to the intended resources, space, or time. For example, the Action 3:30 project trained existing school staff to deliver physical activity programs after school (28–30). The program was highly valued by the school, cost far less than existing provision, and found that children were more active on the days that they attended sessions, with an impact during the after-school period for those who attended. However, challenges within the school in relation to attendance and delivery (context) impacted on the overall efficacy, and the classic trial analysis found no overall difference in children’s physical activity (28–30). This is an example of an intervention that holds considerable promise in theory, but in practice, issues around school setting and delivery diluted the intervention effect and as a result, a potentially very useful program was deemed to be ineffective.

One way that context can impact on school-based research is the provision of outcome data. For example, the adherence to accelerometer wear time protocols has been identified as a moderator of whether a study reported a positive effect, with poor measurement adherence more likely in disadvantaged schools (3). Lower levels of data provision in more deprived areas can mean that a potentially impactful intervention can be missed as there are less data to determine whether or not the intervention was successful. A related issue is that pilot and feasibility studies are often conducted in carefully selected schools that are more supportive of the research process (i.e., a more supportive context), and tend to come from more affluent settings (31) This can result in an over-estimate of any intervention effect (31). Thus, there is a need to understand how context can impact on the data provided.

## 5. Why are current “normal” approaches not working?

We have identified how adherence to intervention fidelity and effectiveness of school-based physical activity interventions differ in different contexts, but context has rarely been considered in the design of physical activity interventions. A key issue here is that researchers often focus on implementing new programs rather than improving programs that already exist within a school, which results in additional content that may have limited buy-in or fit within the context. To improve existing provision, we need approaches that recognize and respond to differences at the school level (school-specific context) which affects both the way in which interventions have been conceptualized and the framework (both design and analysis) used to evaluate the interventions. Each of these is discussed below.

### 5.1. Context in school-based physical activity interventions

Globally, most school-based physical activity interventions have been developed using processes that are consistent with the MRC framework for complex interventions (32). This includes conceiving content based on models of behavior change, intervention mapping, and qualitative research to inform intervention content. New content is then piloted, feasibility studies are conducted and then if there is ‘evidence of promise’, studies progress to a definitive trial, usually a cluster randomized controlled trial (RCT). A key limitation of the way in which these steps have been conducted is that research teams have often paid little attention to the context within which the programs will be delivered.

We recently conducted a systematic review of paired pilot and definitive obesity prevention trials that involved children or young people (≤18 years of age). Most of these studies included a physical activity component. We then looked at whether there was risk of generalizability bias in the paired study (31), that is, features or context of the pilot study that were not generalizable to a full trial. Many of these studies failed to scale when tested in a larger trial as they did not focus on how the studies would be implemented in larger settings or different contexts, and had both delivery agent (delivered by highly competent members of the research team) and implementation support (extra support provided) biases (31). When the results from this review were combined in a meta-analysis, reductions in delivery agent and implementation support were associated with an attenuation of the effect size from pilot study to full trial. Further analysis showed that these biases were particularly likely to have been present in small pilot studies, where implementation biases led to misleadingly positive findings that were not replicated when scaled up in a full trial (31). This often reflects an over-reliance on highly-skilled deliverers who are motivated to see the intervention fully delivered, and these circumstances cannot be replicated in wider practice (33). Similar issues have been reported when attempting to implement programs that have been shown to be effective in ‘efficacy studies’ (34). Conceptually, this work also highlights the difficulty in trying to separate complex interventions from their context. It also raises the question of whether we should even be trying to separate content and context, as interventions are more than the sum of their parts, and the interactions and synergies across parts are likely to be essential for effectiveness. The recently updated MRC guidance for complex interventions highlights that “complexity might also arise through interactions between the intervention and its context” (35). It is therefore important to ensure that any intervention is “fit for purpose” within the context it will be delivered after the research period has ended. In practice this implies that for maximum impact, all school-based interventions should be tailored to the local context.

**Figure 1:**
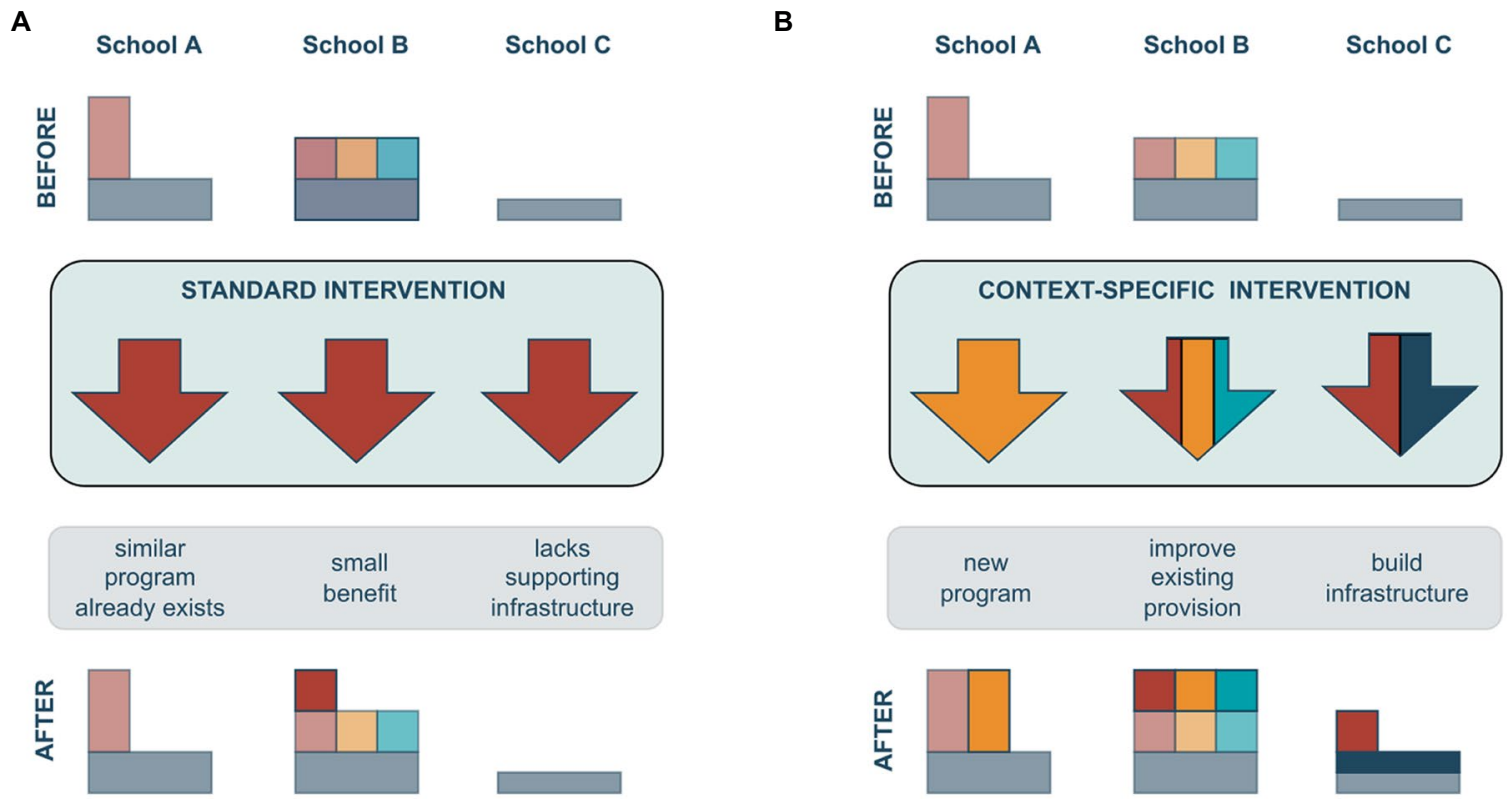
Standard intervention **(A)** compared to context-specific intervention **(B)**. In a standard intervention, each school receives the same intervention, regardless of school-specific context. In a context-specific intervention, the intervention is tailored to be most effective for each specific school.

The current focus is on designing intervention content and ensuring that uniform intervention content is delivered in the same way in each school, regardless of context. Teams then evaluate whether the intervention is delivered as planned, with a focus on internal validity (2, 36). This is unhelpful, as schools may ultimately change some elements to make them work within their setting. As such, it may be more informative to conceptualize intervention components as “essential” and “peripheral.” For example, we might argue that the provision of a new after-school physical activity club is a core component of an intervention but playground markings to support that activity (which may be important for a specific school, and their local context), would be peripheral. If we accept that there will be local adaptation, then identifying the degree of acceptable adaptation for just the essential components, and monitoring what adaptation occurs would be beneficial. This is in contrast to the standard approach of the field where the focus has been on tightly constrained interventions that are not adapted to the local context. This standard approach is not optimal as not only does it not take account of the culture, ethos, priorities, context, and complex systems in each school, but it actively attempts to control these factors to achieve consistency across settings. This results in the estimation of an average treatment effect which is unlikely to reflect un-controlled “real life” effects (Figure 1A). Instead, we argue that the focus should be on creating the best intervention approach for each setting for the agreed outcome of interest, and then seek an evaluation design that facilitates the assessment of the efficacy of that approach.

Understanding rather than ignoring the context is important because some schools will be in affluent, well-resourced areas with financial and in-kind support from parents, local authorities, and third-sector groups, while other schools will be in more economically challenged or rural areas and may have very limited budgets, space or other challenges. These contextual factors have the potential to impact implementation within schools (37) and will shape what kind of physical activity intervention would be appropriate and successful. Schools may already have an initiative that mimics or is identical to an intervention program being offered, and so a “new” program may not be optimized for maximal impact. In other schools, specific activities may not be the best option due to logistical challenges such as space, staff ability or the interests of pupils. A more effective use of resources would be to provide support to extend the current provision. This is consistent with Beets’ Theory of Expanded, Extended, and Enhanced Opportunities (38) which argues that focusing on expanding, extending, and/or enhancing the quality of physical activity provision, depending on the context, are ways to design more effective interventions for increasing physical activity among children within settings. This could be achieved *via* the creation of overall (all schools in a study) and local (school-specific) logic models or program theories to identify how the intervention is intended to operate in different contexts (Figure 1B).

While the importance and role of context has been discussed in the implementation of broader public health interventions (6), context is rarely considered at the earlier stage in designing a physical activity intervention (37). Any new initiative needs to work within existing infrastructure, school policy, staff capacity, pupil needs, and other aspects of the curriculum (39). An approach that works closely with schools, staff, and families from the outset of a project could enable a greater understanding of the particular communities, school culture, and any localized issues which may affect participation in physical activity. This could involve school staff working with pupils to co-create and prioritize activities and then sharing findings with all members of the school community to change the culture in the school. In this approach, there would be a procedure for co-creation that is universal for all schools and includes options provided for potential content. However, the final content in each school is decided upon by the school community. This would result in an individualized intervention at the school level with content tailored to the needs and preference of the school. Time spent building relationships over a long-term and embedding co-production principles such as the sharing of power, including all perspectives and skills, and reciprocity may also improve recruitment and commitment to an intervention (40, 41). This approach would prioritize programs that are consistent with the ethos and priorities of the school and students and thus have greater external validity (38).

### 5.2. Context in the evaluation of interventions

The evaluation of interventions comprises both the evaluation design and analysis methods. These are interlinked, as the study design dictates the available data and in turn the analysis that is possible, while the proposed analysis informs the design. Almost all physical activity interventions in children have been evaluated *via* cluster RCTs (2, 25–27). The cluster RCT design is well-understood and statistically robust, and a pragmatic choice when the intervention is randomly allocated at a group level and the focus is on individual outcomes. In a cluster RCT, context is typically treated as unmeasured factors to be addressed *via* randomization, which creates two interchangeable populations satisfying the conditional exchangeability assumption. However, school context factors occur at cluster-level and in a school-based intervention, there are often comparatively few clusters. While measured and unmeasured confounding factors are balanced between the two groups on average, estimates in any single trial may be far from the truth (42), and school context may differ substantially between control and intervention arms, especially with few clusters. Moreover, in a cluster RCT, unmeasured confounders will be present at both individual and school level. To understand how the effectiveness of interventions depends on different contexts, a cluster RCT should be designed specifically with this aim in mind, to ensure that a suitable range of contexts are included and that the study is powerful enough to allow comparisons between school contexts. This also affects the analysis, as not all standard analysis methods are capable of accounting for non-random differences in school context (for example, marginal models), and those that are will often be underpowered when using standard power calculations. As such, focusing on context may require adaptations to the study design.

School-level variability, which may be indicative of contextual differences, can be considerable and highlights the need to consider context. For example, analysis of the B-Proact1v data showed that between-school variability (attributable to unmeasured school-level factors) accounted for 15% of the total variability (43), compared to just 8% accounted for by key individual variables such as age, gender, and socio-economic position. These unmeasured school factors might include differences in intervention implementation (local context), differences in intervention dose, as well as covariates which differ between schools both randomly (random variation) and systematically (structured variation) such as school policies, ethos, and demographics. School-based physical activity interventions are typically both randomized and applied at school level, which means that school-level factors and between-school variability in outcomes become more relevant; for example, intervention effects can occur at both the individual and group level. This is particularly true if we seek to design interventions that take advantage of the school context, where it is important to understand not just whether an intervention works overall but also how it works, for whom, and in what setting. However, many traditional analysis approaches focus mainly on estimation of the average treatment effect, averaged across all schools (and thus contexts), and are underpowered for estimating school-level heterogeneity (44). Careful planning at the design stage is required to explore contexts where the intervention works well or poorly, for example to identify and collect relevant data, and ensure that there is sufficient variation across schools to enable estimation of differential intervention effects. Without considering such issues at the planning stage, evaluations will have limited ability to explore or fully exploit the context-specific features of an intervention that will best facilitate behavior change.

### 5.3. Outcome measures

The majority of school-based physical activity interventions have focused on the impacts of a program on physical activity and specifically average minutes of MVPA (26). This is driven by national and international physical activity guidelines which recommend that all children and young people engage in an average of 60 min of MVPA per day (7, 8, 14). Many studies are then powered to either detect a difference in the proportion meeting that threshold or a clinically-meaningful difference in mean minutes of MVPA based on a hypothesized change in potential future disease reduction (45–48). It is important to recognize, however, that the physical activity guidelines also recommend the development of motor skills, regular vigorous intensity physical activity and/or activities that develop cardiorespiratory fitness as well as muscle and bone strength. Apart from vigorous intensity physical activity (which can be determined using accelerometers), these elements are hard to quantify and are typically measured *via* self-report surveys. These outcomes and especially cardiorespiratory fitness (49, 50) can be linked to future health, and approaches that focus on improving these outcomes should be encouraged.

Improvements in physical activity during a discrete period, such as physical education lessons, can provide important benefits both in terms of short-term impacts on health and well-being and longer-term motor skill and competency development. These impacts are unlikely to be detectable when conducting a trial to test MVPA that is averaged and therefore attenuated across the week. This is a particular issue as trials of school-based physical activity interventions often have poor compliance with accelerometer protocols (3) and as such we would argue for consideration of a wider range of outcomes in the field. An example of a study with a non-MVPA primary outcome is the Burn 2 Learn RCT which focused on high-intensity activity breaks involving aerobic and resistance exercise in secondary schools (key for muscle and bone strengthening). This study showed that cardiorespiratory and muscular fitness were improved in the intervention group, but there was no impact on accelerometer-assessed average minutes of MVPA (51, 52). Similarly, the Activity and Motivation in Physical Education intervention increased MVPA during physical education lessons (primary outcome) but had no effect on overall MVPA (53), suggesting that a small impact on MVPA during one part of the day can be diluted when looking at averaged MVPA across the entire day. Each of these studies yielded a positive impact on a physical activity related outcome but would have been considered a “failure” if the studies had used the conventional focus on average minutes of MVPA, and highlight the potential utility of a wider set out outcomes.

## 6. Possible solutions

**Figure 2:**
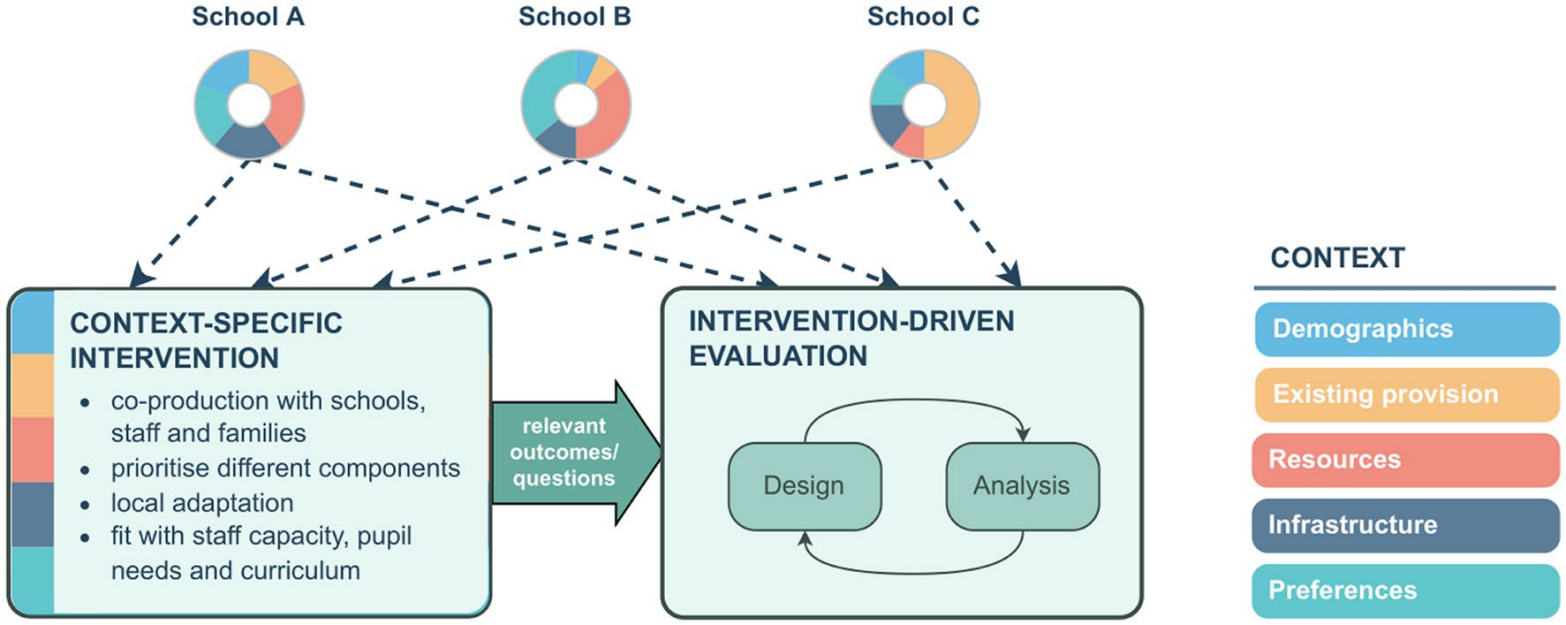
School-specific context feeds into the intervention design, and the intervention informs both the design and analysis.

The evidence presented above highlights a need for a new approach to designing and evaluating school-based physical activity interventions. We have argued firstly that interventions should focus on the context within which an intervention will be delivered (targeting each school’s needs) and secondly that the selected context-specific intervention should dictate the evaluation, rather than vice versa, and ensure that the design can answer relevant questions about relevant outcomes (Figure 2). As a result, we may need to consider alternative interventions, designs, and analyses (35, 54).

### 6.1. Intervention content

A co-produced intervention is an alternative to researcher-controlled and developed intervention content. Researchers could work with staff, pupils, and parents/carers to identify the current strengths and weaknesses of the school physical activity provision, strategies that focus on identified areas for improvement, and then develop components for that priority area (9). This would involve a level of engagement and collaboration with school staff that is consistent with the Creating Active Schools approach (55). Identification of intervention components could be informed by the Consolidated Framework for Implementation Research which encourages researchers to think about: (a) innovation characteristics, such as the adaptability of the “intervention” being implemented; (b) outer setting, such as external polices; (c) inner setting, such as school-specific resources; (d) characteristics of participants, such as pupil demographics and activity levels; and (e) the implementation process, that is, how the program is implemented in each school (10, 37, 56, 57). This approach was used to inform the design of the Burn 2 Learn study highlighted above (58) as well as the iPLAY intervention (59, 60). While this approach would have less consistency between schools and would require time to adopt, implement, and embed new programs, it would have far greater external validity as it reflects actual practice.

### 6.2. Evaluation designs

The evaluation design should be informed by the intervention itself, and one consequence of this is that design and analysis of an evaluation may need to be more complex, which may require additional statistical expertise. RCTs and cluster RCTs are traditionally considered the gold standard of evaluation design (61), and synthesis of studies in the area typically assess study “quality” against risk of bias criteria that are based on key aspects of the RCT design (62–64). However, the trade-off between bias and precision means that estimates are not necessarily more credible than those from any other design (42). As well as other randomized controlled designs, the importance of alternative evaluation designs is recognized in the recently updated UK Medical Research Council guidance for complex interventions which explicitly recognizes the need to consider a wider range of designs than just randomized controlled trials (35), including natural experiment designs (65).

#### 6.2.1. Non-randomized designs

Non-randomized designs (natural experiments/quasi-experimental studies) (66), have the marked advantage that they can evaluate policies or programs as they are implemented in practice and so can provide “real-world” evaluations (67). Quasi-experiments, in which allocation to intervention arm is not randomized by the researcher, facilitate the assessment of whether a program or intervention is implemented and can be particularly useful when randomization is not possible, for example when a program has already been put in place, perhaps in some areas but not others, or when schools choose themselves whether to deliver a program. This therefore has the potential for greater external validity and comparisons can be made by matching to a control group, or estimating a counterfactual. However, as the allocation to intervention is not randomized, there are likely to be confounders that need to be accounted for (68).

Natural experiments are often used to evaluate polices or large-scale structural changes. They include a range of different designs including before and after, difference in differences, interrupted time series and synthetic controls (67). One limitation of natural experiments is that routine data on physical activity outcomes at an appropriate geographical and time scale may not be available. An example of an opportunistic natural experiment is the evaluation of the impact of the 2019 Australian bush fires on children’s physical activity (69), where children were encouraged by local public health officials to limit outdoor physical activity during the fires due to poor air quality. In this study, the authors used data that were already being collected in a cluster RCT (59) to examine the impact of the bushfires on the device-measured physical activity of 8 to 10-year-olds. This design was possible as some children lived in areas that experienced bush fires while others did not. The authors used propensity score matching and a difference-in-differences design to compare those exposed to bush fires and those who were not. The study found that there was little evidence that the targeted public health advice had an impact on the children’s physical activity so the specific intervention of advice during a challenging public health situation had limited impact. This evaluation is an example of how opportunistic natural experiments can answer key policy questions.

#### 6.2.2. Randomized controlled designs

As discussed above, cluster RCTs are commonly used in the evaluation of school-based physical activity interventions, but these have limitations when considering context-specific interventions. The strength of the cluster RCT lies in estimation of the average treatment effect with minimal assumptions, but this is also their limitation when trying to understand how the effectiveness of interventions depends on different contexts as they can identify only the mean of the distribution of treatment effects. In complex situations, we may not be able to guarantee unbiasedness (for example, due to lack of blinding, or differences in the intervention delivery) and may additionally suffer from low precision due to potentially large between-school differences. More complex extensions may require custom power calculations, such as simulation-based methods (70), that focus explicitly on both the number of clusters, as well as the number of individuals. A key issue is that a much larger number of schools will be required compared to a standard cluster RCT design to ensure representation of a good range of contexts in both intervention and control schools. While techniques such as stratification or matching can address baseline differences in context, these are limited to a small number of clearly measurable factors, and so are not useful in isolation. In practice, it is not clear what school context factors affect children’s physical activity, and there are likely to be many interacting factors, including difficult-to-measure factors such as school priorities, attitudes, and culture.

A design where each school acts as its own control would reduce the number of schools needed and maximize the information available on factors associated with the intervention. One example of this is the cluster randomized crossover design, where schools receive both control and intervention, with the order allocated at random (71). However, this is problematic for school-based interventions as it assumes that the intervention has no lasting effects, i.e., no carryover. For a context-specific intervention, this is an important consideration, as the order of intervention/control changes the context.

An alternative, pragmatic design is a cohort-based stepped wedge design in which all schools begin in the control arm and transition in a randomized order from control to intervention (see Supplementary Figure 1) (72). The stepped wedge (and related designs such as the Dynamic Wait-List design (73)) is a form of cross-over trial with randomization in a unidirectional sequence. The repeated measures make it possible to separate within and between-school variability to explore differences between schools and provides considerable statistical efficacy. For example, a standard two-arm cluster RCT with 50:50 randomization to intervention and control results in a quarter of all measurements being post-intervention. By contrast, a stepped wedge design has half of all measurements taken post-intervention and includes repeated measurements from schools. This still allows evaluation of the overall intervention effect but can additionally be used to explore school-specific factors, heterogeneity of treatment effects and change over time. However, because measurements taken under control conditions are systematically earlier than those under intervention conditions, there is greater risk of bias due to confounding by time, such as secular trends over time, between and within school correlations that change over time, and time-varying treatment effects (74). These must be treated analytically and so are at risk of misspecification and result in increased complexity in design, analysis and reporting (75).

## 7. Example: PASSPORT project

The goal of the **PASSPORT** project is to create a physical activity portfolio intervention that is sufficiently flexible to be adapted to the context of each school and includes elements that can be delivered across the school day to maximize the options within a school-specific context. In this approach, component parts of interventions that have shown promise will be identified by key stakeholders at the school (pupils, teachers, parents, and any relevant community groups), resources to deliver the content will be developed, and schools will combine elements to produce their own portfolio of components. A key focus will be adopting an implementation support framework so that the program is ready for dissemination based on the PRACTIS guide (76).

In this project, we are interested in evaluating the overall portfolio approach, i.e., the ability to select components and build something that works for each school, rather than any individual school-specific portfolio. However, we are also interested in how the effectiveness of the intervention varies with different school contexts, over time and in the individual effectiveness of selected core components. In this instance, the outcome of interest could either be average minutes of MVPA across the week or it could be a more context-specific weekday MVPA as the primary outcome with MVPA across the week as an important secondary outcome.

Initial power calculations suggested that a cluster RCT powered to explore all these questions would require an infeasible number of schools. Instead, we chose a stepped wedge design, which can evaluate the overall approach, but also maximizes the ability to explore school context factors, due to more intervention measurements and schools serving as their own control. It also lends itself naturally to looking at change over time. Furthermore, the repeated-measures inherent in the stepped wedge design mean that selected individual intervention components can be analyzed as a multi-arm stepped wedge natural experiment. Conceptually, this is related to a difference-in-differences design, but with the additional stepped wedge structure. We therefore plan to draw on several designs to best address the intervention-specific questions raised above.

## 8. Conclusion

Physical activity is critical for children’s current and future physical and mental health, but current school-based approaches to increasing physical activity have had limited impact. Alternative approaches to both the design and evaluation of school-based interventions are needed. In this paper, we argue that knowledge of the school context is key, and we propose that the field should move away from tightly-constructed interventions that focus on maximizing internal validity, toward a more flexible approach that enables schools to tailor content to their specific setting, and for which the results will be more generalizable. We have also argued that the evaluation of interventions should be driven by the intervention itself, and that cluster RCTs have several limitations for school-specific interventions which depend on the school context. Alternative designs such as natural experiment evaluations and stepped-wedge designs warrant further consideration, as does the use of a wider range of primary outcomes that match the context of the intervention. We accept that the focus on context-specific interventions and evaluation is untested, but we hope that by presenting this argument, we can stimulate a debate of the key issues to improve future physical activity intervention development and implementation.

## Data availability statement

The original contributions presented in the study are included in the article/Supplementary material, further inquiries can be directed to the corresponding author.

## Author contributions

RJ and RS conceived the paper, with ideas developed further by extensive discussions with FdV, MB, DH, DL, and CW. RJ is the study Principal Investigator. RJ and RS wrote the first draft of the paper. All authors reviewed the paper and made key intellectual contributions to content and reporting and approved the final manuscript.

## Funding

This project is funded by UKRI REF EP/X023508/1. RJ and FdV are partly funded by the by the National Institute for Health and Care Research Applied Research Collaboration West (NIHR ARC West). RJ is partly supported by the National Institute for Health and Care Research Bristol Biomedical Research Centre (Bristol BRC). DL is funded a National Health and Medical Research Council Senior Research Fellowship (APP1154507). The views and opinions expressed herein are those of the authors and do not necessarily reflect those of any funder. Funders were not involved in data analysis, data interpretation, or writing of the paper.

## Conflict of interest

The authors declare that the research was conducted in the absence of any commercial or financial relationships that could be construed as a potential conflict of interest.

## Publisher’s note

All claims expressed in this article are solely those of the authors and do not necessarily represent those of their affiliated organizations, or those of the publisher, the editors and the reviewers. Any product that may be evaluated in this article, or claim that may be made by its manufacturer, is not guaranteed or endorsed by the publisher.

## Abbreviations

MVPA: Moderate-to-vigorous intensity physical activity
RCT: Randomized Controlled Trial

## References

[1] Milton K Cavill N Chalkley A Foster C Gomersall S Hagstromer M. Eight investments that work for physical activity. J Phys Act Health. (2021) 18:625–30. doi: 10.1123/jpah.2021-0112, PMID: 33984836

[2] Jones M Defever E Letsinger A Steele J Mackintosh K. A mixed-studies systematic review and meta-analysis of school-based interventions to promote physical activity and/or reduce sedentary time in children. J Sport Health Sci. (2020) 9:3–17. doi: 10.1016/j.jshs.2019.06.009, PMID: 31921476 PMC6943767

[3] Borde R Smith JJ Sutherland R Nathan N Lubans DR. Methodological considerations and impact of school-based interventions on objectively measured physical activity in adolescents: a systematic review and meta-analysis. Obes Rev. (2017) 18:476–90. doi: 10.1111/obr.12517, PMID: 28187241

[4] Pfadenhauer LM Gerhardus A Mozygemba K Lysdahl KB Booth A Hofmann B. Making sense of complexity in context and implementation: the context and implementation of complex interventions (CICI) framework. Implement Sci. (2017) 12:21. doi: 10.1186/s13012-017-0552-5, PMID: 28202031 PMC5312531

[5] Rychetnik L Frommer M Hawe P Shiell A. Criteria for evaluating evidence on public health interventions. J Epidemiol Community Health. (2002) 56:119–27. doi: 10.1136/jech.56.2.119, PMID: 11812811 PMC1732065

[6] Craig P Di Ruggiero E Frohlich KL Mykhalovskiy E White M Campbell R. Taking account of context in population health intervention research: Guidance for producers, users and funders of research. Southampton: NIHR Journal Library (2018).

[7] Bull FC Al-Ansari SS Biddle S Borodulin K Buman MP Cardon G. World Health Organization 2020 guidelines on physical activity and sedentary behaviour. Br J Sports Med. (2020) 54:1451–62. doi: 10.1136/bjsports-2020-102955, PMID: 33239350 PMC7719906

[8] Chaput J Willumsen J Bull FC Chou R Ekelund U Firth J. WHO guidelines on physical activity and sedentary behaviour for children and adolescents aged 5-17 years: summary of the evidence. Int J Behav Nutr Phys Act. (2020) 17:141. doi: 10.1186/s12966-020-01037-z 33239009 PMC7691077

[9] Okely AD Lubans DR Morgan PJ Cotton W Peralta L Miller J. Promoting physical activity among adolescent girls: the girls in sport group randomized trial. Int J Behav Nutr Phys Act. (2017) 14:81. doi: 10.1186/s12966-017-0535-6, PMID: 28637470 PMC5480114

[10] Brophy S Rees A Knox G Baker JS Thomas NE. Child fitness and father’s BMI are important factors in childhood obesity: a school based cross-sectional study. PLoS One. (2012) 7:e36597. doi: 10.1371/journal.pone.0036597, PMID: 22693553 PMC3365059

[11] Parfitt G Eston RG. The relationship between children’s habitual activity level and psychological well-being. Acta Paediatr. (2005) 94:1791–7. doi: 10.1111/j.1651-2227.2005.tb01855.x, PMID: 16421041

[12] Twisk JW Kemper HC van Mechelen W. Tracking of activity and fitness and the relationship with cardiovascular disease risk factors. Med Sci Sports Exerc. (2000) 32:1455–61. doi: 10.1097/00005768-200008000-00014, PMID: 10949012

[13] Aira T Vasankari T Heinonen OJ Korpelainen R Kotkajuuri J Parkkari J. Physical activity from adolescence to young adulthood: patterns of change, and their associations with activity domains and sedentary time. Int J Behav Nutr Phys Act. (2021) 18:85. doi: 10.1186/s12966-021-01130-x, PMID: 34193150 PMC8246658

[14] UK Chief Medical Officers. UK chief medical Officers’ physical activity guidelines. London: Department of Health and Social Care (2019).

[15] Griffiths LJ Cortina-Borja M Sera F Pouliou T Geraci M Rich C. How active are our children? Findings from the millennium cohort study. BMJ Open. (2013) 3:e002893. doi: 10.1136/bmjopen-2013-002893, PMID: 23965931 PMC3752053

[16] Cooper AR Goodman A Page AS Sherar LB Esliger DW van Sluijs EM. Objectively measured physical activity and sedentary time in youth: the international children’s accelerometry database (ICAD). Int J Behav Nutr Phys Act. (2015) 12:113. doi: 10.1186/s12966-015-0274-5, PMID: 26377803 PMC4574095

[17] Nader PR Bradley RH Houts RM McRitchie SL O’Brien M. Moderate-to-vigorous physical activity from ages 9 to 15 years. JAMA. (2008) 300:295–305. doi: 10.1001/jama.300.3.295 18632544

[18] Stockwell S Trott M Tully M Shin J Barnett Y Butler L. Changes in physical activity and sedentary behaviours from before to during the COVID-19 pandemic lockdown: a systematic review. BMJ Open Sport Exerc Med. (2020):7. doi: 10.1136/bmjsem-2020-000960 PMC7852071 34192010

[19] Paterson DC Ramage K Moore SA Riazi N Tremblay MS Faulker G. Exploring the impact of COVID-19 on the movement behaviors of children and youth: a scoping review of evidence after the first year. J Sport Health Sci. (2021) 10:675–89. 34237456 10.1016/j.jshs.2021.07.001 PMC8687706

[20] Office for Health Improvement and Disparities. COVID-19 mental health and wellbeing surveillance: Report In: Office for Health Improvement and Disparities. London: OHID (2022)

[21] Racine N McArthur BA Cooke JE Eirich R Zhu J Madigan S. Global prevalence of depressive and anxiety symptoms in children and adolescents during COVID-19: a meta-analysis. JAMA Pediatr. (2021) 175:1142–50. doi: 10.1001/jamapediatrics.2021.2482, PMID: 34369987 PMC8353576

[22] Salway R Foster C de Vocht F Tibbitts B Emm-Collison L House D. Accelerometer-measured physical activity and sedentary time among children and their parents in the UK before and after COVID-19 lockdowns: a natural experiment. Int J Behav Nutr Phys Act. (2022) 19:51. doi: 10.1186/s12966-022-01290-4, PMID: 35570265 PMC9107948

[23] Sport England. The impact of coronavirus on activity levels revealed. London: Sport England (2021).

[24] Jago R Baranowski T. Non-curricular approaches for increasing physical activity in youth: a review. Prev Med. (2004) 39:157–63. doi: 10.1016/j.ypmed.2004.01.014, PMID: 15207997

[25] Love R Adams J van Sluijs EMF. Are school-based physical activity interventions effective and equitable? A systematic review and meta-analysis of cluster randomised controlled trials. Lancet. (2018) 392:S53. doi: 10.1016/S0140-6736(18)32174-3 PMC6563481 30628172

[26] Love R Adams J van Sluijs EMF. Are school-based physical activity interventions effective and equitable? A meta-analysis of cluster randomized controlled trials with accelerometer-assessed activity. Obes Rev. (2019) 20:859–70. doi: 10.1111/obr.12823, PMID: 30628172 PMC6563481

[27] Love R Adams J van Sluijs EMF. Equity effects of children’s physical activity interventions: a systematic scoping review. Int J Behav Nutr Phys Act. (2017) 14:314. doi: 10.1186/s12966-017-0586-8 PMC5625682 28969638

[28] Jago R Sebire SJ Davies B Wood L Edwards MJ Banfield K. Randomised feasibility trial of a teaching assistant led extracurricular physical activity intervention for 9 to 11 year olds: action 3:30. Int J Behav Nutr Phys Act. (2014) 11:114. doi: 10.1186/s12966-014-0114-z, PMID: 25209323 PMC4172904

[29] Jago R Tibbitts B Porter A Sanderson E Bird E Powell JE. A revised teaching assistant-led extracurricular physical activity programme for 8- to 10-year-olds: the action 3:30R feasibility cluster RCT. Public Health Res. (2019) 7:1–128. doi: 10.3310/phr07190 31869014

[30] Jago R Tibbitts B Sanderson E Bird EL Porter A Metcalfe C. Action 3:30R: results of a cluster randomised feasibility study of a revised teaching assistant-led extracurricular physical activity intervention for 8 to 10 year olds. Int J Environ Res Public Health. (2019) 16:131. doi: 10.3390/ijerph16010131, PMID: 30621326 PMC6339197

[31] Beets MW Weaver RG Ioannidis JPA Geraci M Brazendale K Decker L. Identification and evaluation of risk of generalizability biases in pilot versus efficacy/effectiveness trials: a systematic review and meta-analysis. Int J Behav Nutr Phys Act. (2020) 17:19. doi: 10.1186/s12966-020-0918-y, PMID: 32046735 PMC7014944

[32] Craig P Dieppe P Macintyre S Michie S Nazareth I Petticrew M. Developing and evaluating complex interventions: the new Medical Research Council guidance. BMJ. (2008) 337:a1655. doi: 10.1136/bmj.a1655 18824488 PMC2769032

[33] Lane C McCrabb S Nathan N Naylor PJ Bauman A Milat A. How effective are physical activity interventions when they are scaled-up: a systematic review. Int J Behav Nutr Phys Act. (2021) 18:16. doi: 10.1186/s12966-021-01080-4, PMID: 33482837 PMC7821550

[34] Milat AJ Bauman A Redman S. Narrative review of models and success factors for scaling up public health interventions. Implement Sci. (2015) 10:113. doi: 10.1186/s13012-015-0301-6, PMID: 26264351 PMC4533941

[35] Skivington K Matthews L Simpson SA Craig P Baird J Blazeby JM. A new framework for developing and evaluating complex interventions: update of Medical Research Council guidance. BMJ. (2021) 374:n 2061. doi: 10.1136/bmj.n2061 PMC8482308 34593508

[36] Keshavarz N Nutbeam D Rowling L Khavarpour F. Schools as social complex adaptive systems: a new way to understand the challenges of introducing the health promoting schools concept. Soc Sci Med. (2010) 70:1467–74. doi: 10.1016/j.socscimed.2010.01.034, PMID: 20207059

[37] McHale F Ng K Scanlon D Cooper J Grady C Norton C. Implementation evaluation of an Irish secondary-level whole school programme: a qualitative inquiry. Health Promot Int. (2022) 37:daac131. doi: 10.1093/heapro/daac131, PMID: 36287522

[38] Beets MW Okely A Weaver RG Webster C Lubans D Brusseau T. The theory of expanded, extended, and enhanced opportunities for youth physical activity promotion. Int J Behav Nutr Phys Act. (2016) 13:120. doi: 10.1186/s12966-016-0442-2, PMID: 27852272 PMC5112641

[39] Woods CB Volf K Kelly L Casey B Gelius P Messing S. The evidence for the impact of policy on physical activity outcomes within the school setting: a systematic review. J Sport Health Sci. (2021) 10:263–76. doi: 10.1016/j.jshs.2021.01.006, PMID: 33482424 PMC8167338

[40] Hickey G Brearley S Coldham T Denegri S Green G Staniszewska S. NIHR involve: guidance on co-producing a research project. Southampton: INVOLVE (2019).

[41] Farr M Davies R Davies P Bagnall D Branagan E Andrews H. A map of resources for co-producing research in health and social care. Bristol, UK: National Institute for Health Research (NIHR) ARC west and people in health west of England (2020).

[42] Deaton A Cartwright N. Understanding and misunderstanding randomized controlled trials. Soc Sci Med. (2018) 210:2–21. doi: 10.1016/j.socscimed.2017.12.005, PMID: 29331519 PMC6019115

[43] Salway R Emm-Collison L Sebire SJ Thompson JL Lawlor DA Jago R. A multilevel analysis of Neighbourhood, school, friend and individual-level variation in primary school Children’s physical activity. Int J Environ Res Public Health. (2019) 16:4889. doi: 10.3390/ijerph16244889, PMID: 31817182 PMC6950546

[44] Yang S Li F Starks MA Hernandez AF Mentz RJ Choudhury KR. Sample size requirements for detecting treatment effect heterogeneity in cluster randomized trials. Stat Med. (2020) 39:4218–37. doi: 10.1002/sim.8721, PMID: 32823372 PMC7948251

[45] Harrington D Davies MJ Bodicoat D Charles JM Chudasama YV Gorely T. A school-based intervention (‘girls active’) to increase physical activity levels among 11- to 14-year-old girls: cluster RCT. Southampton, UK: NIHR Journals Library (2019). 7 p. 30779533

[46] Corder K Sharp SJ Jong ST Foubister C Brown HE Wells EK. Effectiveness and cost-effectiveness of the GoActive intervention to increase physical activity among UK adolescents: a cluster randomised controlled trial. PLoS Med. (2020) 17:e1003210. doi: 10.1371/journal.pmed.1003210, PMID: 32701954 PMC7377379

[47] Willis K Tibbitts B Sebire SJ Reid T MacNeill SJ Sanderson E. Protocol for a cluster randomised controlled trial of a peer-led physical activity iNtervention for adolescent girls (PLAN-A). BMC Public Health. (2019) 19:644. doi: 10.1186/s12889-019-7012-x, PMID: 31138171 PMC6537278

[48] Sebire SJ Edwards MJ Campbell R Jago R Kipping R Banfield K. Protocol for a feasibility cluster randomised controlled trial of a peer-led school-based intervention to increase the physical activity of adolescent girls (PLAN-A). Pilot Feasibility Stud. (2016) 2:2. doi: 10.1186/s40814-015-0045-8, PMID: 27966675 PMC4770840

[49] Garcia-Hermoso A Ramirez-Velez R Garcia-Alonso Y Alonso-Martinez AM Izquierdo M. Association of Cardiorespiratory Fitness Levels during Youth with Health Risk Later in life: a systematic review and meta-analysis. JAMA Pediatr. (2020) 174:952–60. doi: 10.1001/jamapediatrics.2020.2400, PMID: 32870243 PMC7489376

[50] Raghuveer G Hartz J Lubans DR Takken T Wiltz JL Mietus-Snyder M. Cardiorespiratory fitness in youth: an important marker of health: a scientific statement from the American Heart Association. Circulation. (2020) 142:e101–18. doi: 10.1161/CIR.0000000000000866, PMID: 32686505 PMC7524041

[51] Lubans DR Smith JJ Eather N Leahy AA Morgan PJ Lonsdale C. Time-efficient intervention to improve older adolescents’ cardiorespiratory fitness: findings from the ‘Burn 2 Learn’ cluster randomised controlled trial. Br J Sports Med. (2020) 10.1136/bjsports-2020-103277 PMC8223670 33355155

[52] Kennedy SG Smith JJ Morgan PJ Peralta LR Hilland TA Eather N. Implementing resistance training in secondary schools: a cluster randomized controlled trial. Med Sci Sports Exerc. (2018) 50:62–72. doi: 10.1249/MSS.0000000000001410, PMID: 29251687

[53] Lonsdale C Lester A Owen KB White RL Peralta L Kirwan M. An internet-supported school physical activity intervention in low socioeconomic status communities: results from the activity and motivation in physical education (AMPED) cluster randomised controlled trial. Br J Sports Med. (2019) 53:341–7. doi: 10.1136/bjsports-2017-097904, PMID: 28993404

[54] Ogilvie D Adams J Bauman A Gregg EW Panter J Siegel KR. Using natural experimental studies to guide public health action: turning the evidence-based medicine paradigm on its head. J Epidemiol Community Health. (2020) 74:203–8. doi: 10.1136/jech-2019-213085, PMID: 31744848 PMC6993029

[55] Daly-Smith A Quarmby T Archbold VSJ Corrigan N Wilson D Resaland GK. Using a multi-stakeholder experience-based design process to co-develop the creating active schools framework. Int J Behav Nutr Phys Act. (2020) 17:13. doi: 10.1186/s12966-020-0917-z, PMID: 32028968 PMC7006100

[56] Damschroder LJ Aron DC Keith RE Kirsh SR Alexander JA Lowery JC. Fostering implementation of health services research findings into practice: a consolidated framework for advancing implementation science. Implement Sci. (2009) 4:50. doi: 10.1186/1748-5908-4-50, PMID: 19664226 PMC2736161

[57] Damschroder LJ Reardon CM Opra Widerquist MA Lowery J. The updated consolidated framework for implementation research based on user feedback. Implement Sci. (2022) 17:75. doi: 10.1186/s13012-022-01245-0 36309746 PMC9617234

[58] Kennedy SG Leahy AA Smith JJ Eather N Hillman CH Morgan PJ. Process evaluation of a school-based high-intensity interval training program for older adolescents: the burn 2 learn cluster randomised controlled trial. Children (Basel). (2020) 7:299. doi: 10.3390/children7120299 33339356 PMC7765884

[59] Lonsdale C Sanders T Parker P Noetel M Hartwig T Vasconcellos D. Effect of a scalable school-based intervention on cardiorespiratory fitness in children: a cluster randomized clinical trial. JAMA Pediatr. (2021) 175:680–8. doi: 10.1001/jamapediatrics.2021.0417, PMID: 33938946 PMC8094033

[60] Lubans DR Sanders T Noetel M Parker P McKay H Morgan PJ. Scale-up of the internet-based professional learning to help teachers promote activity in youth (iPLAY) intervention: a hybrid type 3 implementation-effectiveness trial. Int J Behav Nutr Phys Act. (2022) 19:141. doi: 10.1186/s12966-022-01371-4, PMID: 36451168 PMC9713961

[61] Cartwright N. Are RCTs the gold standard? Bio Societies. (2007) 2:11–20. doi: 10.1017/S1745855207005029

[62] Hollis JL Sutherland R Williams AJ Campbell E Nathan N Wolfenden L. A systematic review and meta-analysis of moderate-to-vigorous physical activity levels in secondary school physical education lessons. Int J Behav Nutr Phys Act. (2017) 14:52. doi: 10.1186/s12966-017-0504-0, PMID: 28438171 PMC5402678

[63] Neil-Sztramko SE Caldwell H Dobbins M. School-based physical activity programs for promoting physical activity and fitness in children and adolescents aged 6 to 18. Cochrane Database Syst Rev. (2021) 9:CD007651. doi: 10.1002/14651858.CD007651 34555181 PMC8459921

[64] Sterne JAC Savović J Page MJ Elbers RG Blencowe NS Boutron I. RoB 2: a revised tool for assessing risk of bias in randomised trials. BMJ. (2019) 366:l 4898. doi: 10.1136/bmj.l4898 31462531

[65] Craig P Campbell M Bauman A Deidda M Dundas R Fitzgerald N. Making better use of natural experimental evaluation in population health. BMJ. (2022) 379:e070872. doi: 10.1136/bmj-2022-070872 36280251 PMC7613963

[66] de Vocht F Katikireddi SV McQuire C Tilling K Hickman M Craig P. Conceptualising natural and quasi experiments in public health. BMC Med Res Methodol. (2021) 21:32. doi: 10.1186/s12874-021-01224-x, PMID: 33573595 PMC7879679

[67] Craig P Cooper C Gunnell D Haw S Lawson K Macintyre S. Using natural experiments to evaluate population health. London: Medical Research Council (2014). 10.1136/jech-2011-200375 PMC3796763 22577181

[68] Barnighausen T Rottingen JA Rockers P Shemilt I Tugwell P. Quasi-experimental study designs series-paper 1: introduction: two historical lineages. J Clin Epidemiol. (2017) 89:4–11. doi: 10.1016/j.jclinepi.2017.02.020, PMID: 28694121

[69] Del Pozo CB Hartwig TB Sanders T Noetel M Parker P Antczak D. The effects of the Australian bushfires on physical activity in children. Environ Int. (2021) 146:106214. doi: 10.1016/j.envint.2020.106214 33157378

[70] Arnold BF Hogan DR Colford JM Jr Hubbard AE. Simulation methods to estimate design power: an overview for applied research. BMC Med Res Methodol. (2011) 11:94. doi: 10.1186/1471-2288-11-94, PMID: 21689447 PMC3146952

[71] Arnup SJ McKenzie JE Hemming K Pilcher D Forbes AB. Understanding the cluster randomised crossover design: a graphical illustraton of the components of variation and a sample size tutorial. Trials. (2017) 18:381. doi: 10.1186/s13063-017-2113-2, PMID: 28810895 PMC5557529

[72] Hussey MA Hughes JP. Design and analysis of stepped wedge cluster randomized trials. Contemp Clin Trials. 28:182–91. 16829207 10.1016/j.cct.2006.05.007

[73] Wyman PA Henry D Knoblauch S Brown CH. Designs for testing group-based interventions with limited numbers of social units: the dynamic wait-listed and regression point displacement designs. Prev Sci. (2015) 16:956–66. doi: 10.1007/s11121-014-0535-6, PMID: 25481512 PMC4458455

[74] Hemming K Taljaard M. Reflection on modern methods: when is a stepped-wedge cluster randomized trial a good study design choice? Int J Epidemiol. (2020) 49:1043–52. doi: 10.1093/ije/dyaa077, PMID: 32386407 PMC7394949

[75] Li F Hughes JP Hemming K Taljaard M Melnick ER Heagerty PJ. Mixed-effects models for the design and analysis of stepped wedge cluster randomized trials: an overview. Stat Methods Med Res. (2021) 30:612–39. doi: 10.1177/0962280220932962, PMID: 32631142 PMC7785651

[76] Koorts H Eakin E Estabrooks P Timperiod A Slamon J Bauman A. Implementation and scale up of population physical activity interventions for clinical and community settings: the PRACTIS guide. Int J Behav Nutr Phys Act. (2018) 15:51. doi: 10.1186/s12966-018-0678-0 29884236 PMC5994105

