# Supplementary material for: Rethinking children’s physical activity interventions at school: A new context-specific approach
Source: Front Public Health. 2023 Apr 13;11:1149883. doi: 10.3389/fpubh.2023.1149883 (PMC10133698; doi:10.3389/fpubh.2023.1149883)
Supplement: Supplementary file 1 [file Table_1.DOCX]

Supplementary Material

Rethinking children’s physical activity interventions at school: a new context-specific approach

Jago R^*^, Salway R, House D, Beets MW, Lubans DR, Woods C, de Vocht F

*** Correspondence:** Prof Russ Jago: [russ.jago@bristol.ac.uk](mailto:russ.jago@bristol.ac.uk)

# Supplementary Figures

**Supplementary Figure 1.** **Comparison of classic cluster randomized controlled trial and Stepped Wedge design**

**
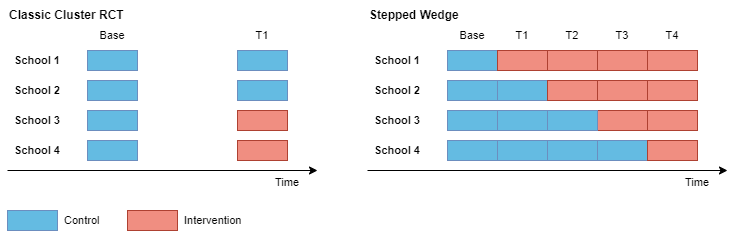
**

.
